# Clinical lipidomics: realizing the potential of lipid profiling

**DOI:** 10.1016/j.jlr.2021.100127

**Published:** 2021-09-25

**Authors:** Thomas G. Meikle, Kevin Huynh, Corey Giles, Peter J. Meikle

**Affiliations:** 1 Metabolomics Laboratory, Baker Heart and Diabetes Institute, Melbourne, Victoria, Australia; 2 Baker Department of Cardiometabolic Health, University of Melbourne, Parkville, Victoria, Australia; 3 Faculty of Medicine, Nursing and Health Sciences, Central Clinical School, Monash University, Melbourne, Victoria, Australia

**Keywords:** clinical lipidomics platform, risk assessment, biomarkers, cardiovascular disease, clinical translation, bioinformatics, epidemiology, diabetes, Alzheimer's disease, cancer, ITSD, internal standard, LASSO, least absolute shrinkage and selection operator, LIPID, Long-Term Intervention with Pravastatin in Ischemic Disease, LIPID MAPS, Lipid Metabolites and Pathways Strategy, NIST, National Institute of Standards and Technology, PC, phosphatidylcholine, QqTOF, quadrupole TOF, SRM, Standard Reference Material

## Abstract

Dysregulation of lipid metabolism plays a major role in the etiology and sequelae of inflammatory disorders, cardiometabolic and neurological diseases, and several forms of cancer. Recent advances in lipidomic methodology allow comprehensive lipidomic profiling of clinically relevant biological samples, enabling researchers to associate lipid species and metabolic pathways with disease onset and progression. The resulting data serve not only to advance our fundamental knowledge of the underlying disease process but also to develop risk assessment models to assist in the diagnosis and management of disease. Currently, clinical applications of in-depth lipidomic profiling are largely limited to the use of research-based protocols in the analysis of population or clinical sample sets. However, we foresee the development of purpose-built clinical platforms designed for continuous operation and clinical integration—assisting health care providers with disease risk assessment, diagnosis, and monitoring. Herein, we review the current state of clinical lipidomics, including the use of research-based techniques and platforms in the analysis of clinical samples as well as assays already available to clinicians. With a primary focus on MS-based strategies, we examine instrumentation, analysis techniques, statistical models, prospective design of clinical platforms, and the possible pathways toward implementation of clinical lipidomics.

Lipid species play an essential role within energy metabolism and cell signaling, as well as serving as the constituent components of plasma membranes and lipid particles such as lipoproteins and extracellular vesicles. Dysregulation of lipid metabolism is closely associated with the initiation and progression of many disease states, including CVD (1), type 2 diabetes (2), Alzheimer's disease (3), and several forms of cancer (4, 5, 6). As such, large-scale experimental analysis of the human lipidome reveals a trove of useful information, guiding our understanding of fundamental biological processes, as well as providing insight into disease cause and progression. Notably, lipidomic profiling captures the biochemical effects of both genetic and lifestyle influences, making the practice particularly useful in relation to complex diseases.

Clinical lipidomics is an emerging subfield that aims to provide clinical and consumer access to lipidomic analysis, the results of which can then be used to predict, diagnose, and manage disease (and health). For the past 60 years, the mainstay of lipid measures employed within a clinical setting has consisted of total triglycerides, total cholesterol, LDL-C, and HDL-C. Whilst the association of these measurements with the development and progression of disease and their utility in diagnosis is well established, these few measurements are poorly descriptive of the total lipidomic profile. Thus, there remains a wealth of metabolic information, which is presently underutilized in the clinical setting.

Current research-based lipidomic platforms are capable of identifying and measuring blood plasma levels of hundreds of different lipid species from dozens of different classes and subclasses. These measurements can be achieved with high throughput, using small volumes of serum or plasma—a development that has enabled detailed lipidomic profiling in several large-scale longitudinal population studies and the retrospective analyses of clinical trials.

Despite the vast amount of metabolic information contained within a lipidomic profile, there remains a large disparity between the capabilities of research-based lipidomics and currently available clinical lipidomic assays. Increasingly, efforts are being made within the international research community—including our own research group—to translate these research protocols to the clinical setting. One of the most promising aspects of these efforts is the use of statistical analysis, epidemiological modeling, and machine learning to develop predictive models that help identify at-risk individuals and stratify risk in those already diagnosed with a disease.

Herein, we review the current state of clinical lipidomics, including the use of research-based techniques and platforms in the analysis of clinical trial data as well as assays already available to clinicians. With a primary focus on MS-based strategies, we examine instrumentation, analysis techniques, prospective design of clinical platforms, and the possible pathways toward implementation of clinical lipidomics.

## Progress toward clinical lipidomics

**Fig. 1:**
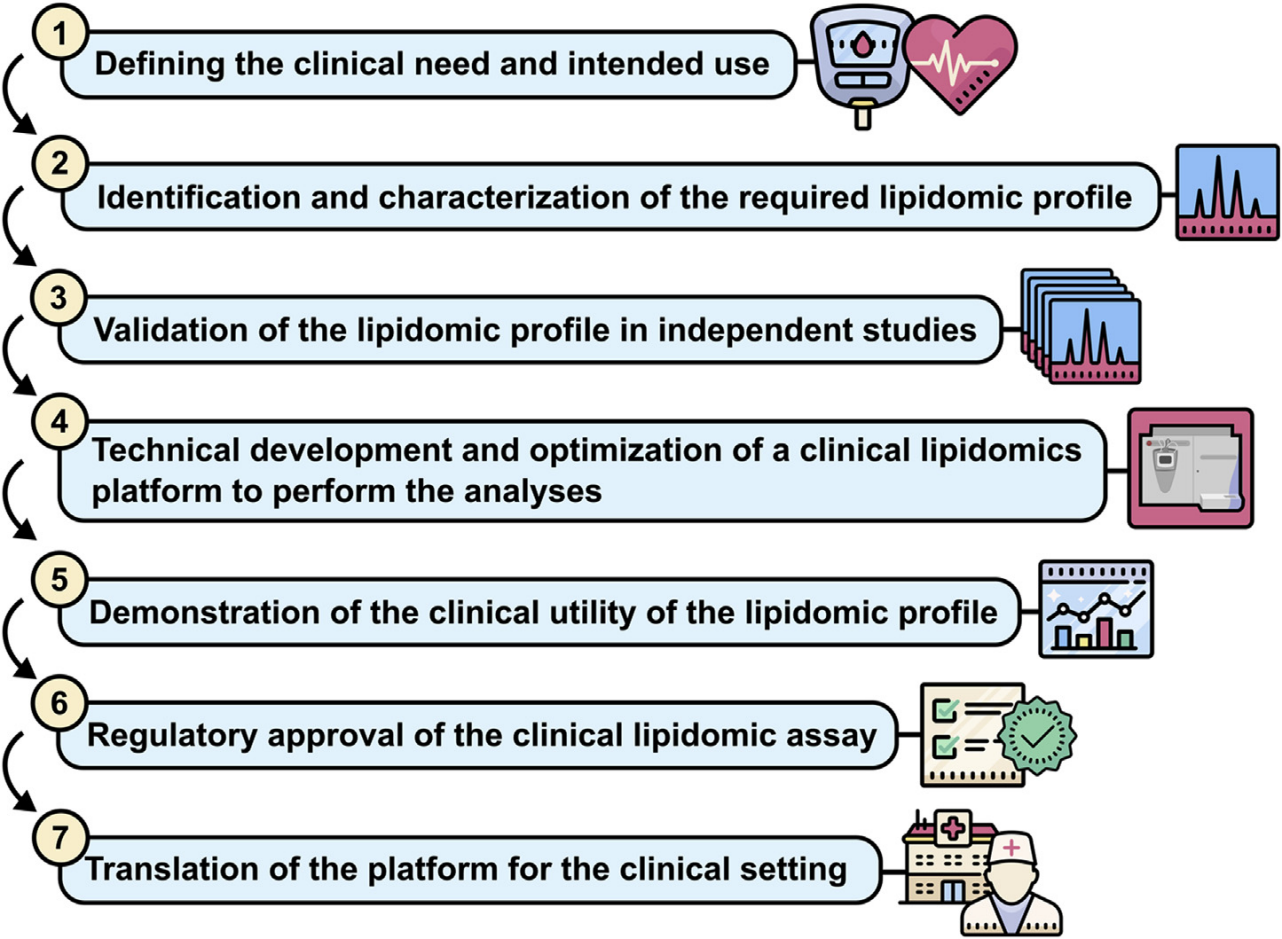
An outline of the key steps in the establishment of a new clinical lipidomics platform.

The ultimate goal of clinical lipidomics is to establish new lipidomic assays, which inform on clinical outcomes. However, the path toward development and translation is complex. For example, consider the following outline of the key steps in the establishment of a new clinical lipidomics platform (Fig. 1).

Despite the numerous hurdles, we are beginning to see the emergence of lipidomic-based clinical diagnostic tools. One notable example is the progressive development of a ceramide assay for the prediction of cardiovascular risk. This assay was born out of work from Laaksonen *et al.* (7), who initially demonstrated that serum ceramide species predict cardiovascular death in patients with stable coronary heart disease and acute coronary syndrome, suggesting these lipids may serve as potential risk biomarkers. The authors subsequently published a protocol for a high-throughput ceramide assay using a 96-well plate format, with analysis carried out via LC-MS/MS, followed by validation of the ceramide association across several different cohorts (8, 9) and the translation of the assay into clinical practice at the Mayo Clinic (10).

While it is of fundamental interest to highlight specific lipids with strong correlations to disease outcomes, for the purposes of clinical lipidomics, predictive statistical models that incorporate numerous lipid species have generally proven more robust to validation across external cohorts. More recently, Hilvo *et al.* (11) developed a model (CERT2) incorporating ceramides with known phosphatidylcholine (PC) cardiovascular risk markers. Their model was developed using lipidomic analysis of the Western Norway Coronary Angiography Cohort (N = 3,789) cohort and validated in two additional studies: the LIPID (Long-Term Intervention with Pravastatin in Ischemic Disease, N = 5,991) trial and Langzeiterfolge der KARdiOLogischen Anschlussheilbehandlung (N = 1,023). The CERT2 score was highly significant in predicting CVD mortality, yielding hazard ratios of 1.44 (1.28–1.63) in The Western Norway Coronary Angiography Cohort, 1.47 (1.34–1.61) in the LIPID trial, and 1.69 (1.31–2.17) in Langzeiterfolge der KARdiOLogischen Anschlussheilbehandlung. The model was further improved via the addition of the nonlipidomic measure, high-sensitivity troponin T. This technology was licensed to Quest Diagnostics for further development into a clinical assay earlier this year.

Recently, we performed in-depth lipidomic analysis of the Action in Diabetes and Vascular Disease: Preterax and Diamicron-MR Controlled Evaluation (N = 3,779) trial, which monitored cardiovascular outcomes in patients with type 2 diabetes, and the LIPID trial, which monitored stable patients with CVD who had suffered a previous acute coronary syndrome (2, 12). We found that future cardiovascular events and death were strongly linked to plasma lipid species, including sphingolipids, phospholipids, cholesteryl esters, and glycerolipids. The resulting lipidomic data were used to create statistical models for the prediction of cardiovascular outcomes, incorporating lipid measures (4–7 species) with traditional risk factors. Validation of these models across trials (as well as robust intra-trial cross-validation) demonstrated significant improvements in the prediction of CVD events and death over traditional risk factors alone.

In addition to assessing risk of future disease, lipid biomarkers have also been proposed to monitor the effects of interventions. The aforementioned LIPID trial was used to identify biomarkers that predicted the risk reduction resulting from statin use (13). Pravastatin was associated with significant changes in the majority of lipid species and classes. A ratio of two lipid species, a phosphatidylinositol (36:2) and a PC (38:4), was identified to be predictive of those who did or did not receive a risk reduction from statin intervention, independent of changes in total cholesterol, HDL-C, and triglycerides. This highlighted a potential biomarker for monitoring of statin treatment and identified novel metabolic pathways involved in the beneficial effects of pravastatin.

### NMR-based clinical lipidomics

While this review focuses primarily on the clinical applications of MS-based lipidomics, it is worth mentioning a number of NMR techniques, which excel where MS-based techniques do not. These techniques exploit the observation that ^1^H NMR signals from terminal methyl groups in lipid hydrocarbon chains of lipoprotein complexes systematically shift to lower frequencies as particle size decreases (14, 15). Otvos *et al.* (16) used a linear least-squares deconvolution process to derive the individual contributions of different lipoprotein subfractions to a given plasma sample's methyl signal, the amplitudes of which can then be used to determine concentration. This method was subsequently developed into a commercial assay, LipoProfile, in 1997 by LipoScience. LipoProfile is capable of simultaneously quantifying VLDL, LDL, and HDL into 11 lipoprotein subclasses from a single measurement, acquired in approximately 1 min. LipoScience went on to develop a NMR-based commercial LDL-P (particle number) diagnostic platform, the Vantera® Clinical Analyzer, which was cleared for market by the Food and Drug Administration in 2013.

Soininen *et al.* (17, 18, 19, 20) developed an assay specifically designed for clinical use, incorporating NMR measurements across three molecular windows; one for lipoprotein analysis; a second using a T2-relaxation–filtered pulse sequence to suppress the macromolecule and lipoprotein signals and improve the detection of low molecular weight metabolites; and a third for serum lipid extracts (17, 18, 19, 20). As of 2015, they claim an analysis time of ∼5 min per sample, with a possible throughput of ∼80,000 samples annually (18). These techniques have been employed by Finnish company, Nightingale, in the development of a commercial platform, marketed for deployment in large-scale clinical trials and as a personal health measure. Measurements of HDL and LDL particle number have proven to be superior indicators of cardiovascular risk than traditional total cholesterol measurements (21, 22, 23, 24). However, the major limitation of NMR-based techniques is the lack of resolution at the individual lipid species level, because of the complex nature of the sample matrix.

## Bioinformatics, risk model development, and evaluation

Much of the utility offered by clinical lipidomics stems from the application of statistical modeling to provide accurate prediction of patient risk, aiding in the diagnosis of disease, and enabling a personalized approach to treatments and interventions. The clinically focused lipidomic studies cited herein typically adopt one of several feature selection strategies to yield predictive models with a minimal number of lipid species. These parsimonious models are desirable to facilitate translation into a clinical setting. The measurement of fewer lipids potentially enables the use of stable isotope internal standards for each lipid species, leading to improved assay performance. Common feature selection strategies include forward/backward stepwise selection, based on some performance metric such as area under the receiver operating characteristic curve or Akaike's information criteria, and least absolute shrinkage and selection operator (LASSO) regression. These latter approaches are typically performed within a cross-validated framework to provide estimates of internal validation; however, all models would ideally be validated on independent cohorts. For a comprehensive review on model development, optimization, and validation of lipidomic models, see the study by Giles *et al.* (25).

Lipid ratios are an attractive option for risk model development because of their simplicity and their inherent ability to provide internal normalization of the lipidomic data, thereby controlling unwanted variation (7, 9). These lipid ratios typically contain two biologically relevant lipids that show opposing associations with the outcome of interest and thus strengthen the association and predictive performance. The challenge with these models, which might contain up to 20 lipid species, is the optimal selection of lipids because of the strong correlation structure within the lipidome (2, 12, 26).

**Fig. 2:**
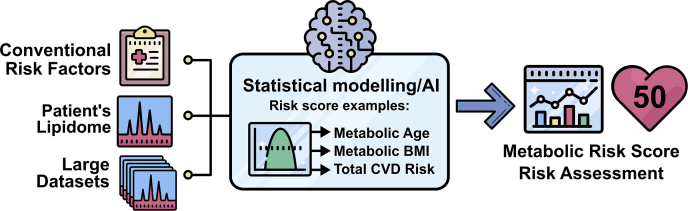
A diagrammatic flowchart illustrating the generation of risk scores from lipidomic data and conventional risk factors.

More recently, it has been recognized that complex models containing larger numbers of lipids may offer some advantages. Such models may contain lipid species numbering from dozens to several hundred, many of which may be correlated. Statistical models, such as Ridge regression, allow the creation of robust risk models in the presence of high collinearity. This approach can produce models that are better able to capture the complexity of lipid metabolism and may also improve model stability across cohorts or populations. A recent approach for creating such models is the prediction of physiological or clinical risk traits, such as age and BMI, using a patient's lipidomic profile, illustrated in Fig. 2. The resulting *metabolic* BMI, for instance, should then capture individual variation in the metabolic state above that captured by the BMI measure itself. It is anticipated that such measures may then provide improved performance in the prediction of disease outcomes above the measured values. This approach was utilized by Gerl *et al.* when they performed modeling of the lipidome (183 plasma lipid species) to predict BMI, waist circumference, and body fat percentage using multiple approaches, including random forest, stochastic gradient boosting, partial least squares, LASSO, and Cubist models, with the latter two showing the best results. The best performing LASSO model explained 73% of body fat percentage with a model utilizing 58 lipid species (27).

Evaluation of the additive benefit of lipidomic risk models above traditional risk markers has also been a topic of considerable interest. This is usually achieved by adding lipid species to an existing set of risk markers and then assessing the improvement in the model performance by metrics such as area under the receiver operating characteristic curve, Akaike's information criteria, *R*^2^, or percentage accuracy. It is important to recognize that including additional variables in a regression model will always result in an improved apparent performance, for many of the aforementioned performance metrics. Cross-validation can offer evidence that the improvement in performance is generalizable. These metrics do not always capture the clinical benefit of alternative models. Therefore, alternative performance metrics, such as the continuous or categorical net reclassification index and integrated discrimination improvement metrics have also been used. While debate continues over the best approach (28), it will be important for any new test to provide some comparison to existing measures and clearly demonstrate an improvement in the clinical utility of lipidomic tests over existing measures. Equally important will be the ability to obtain regulatory approval for these new tests which, with a large number of individual lipid species, will not easily fall within the current regulatory framework in many countries.

## Clinical platform design

### Lipid classification and pathways

The enormous structural diversity of lipids has implications for the analysis of complex biological samples, including applicable instrumentation and analytical techniques. The Lipid Metabolites and Pathways Strategy (LIPID MAPS), established with the aim of advancing lipidomics practices, proposed the classification of lipids into eight categories: fatty acyls, glycerolipids, glycerophospholipids, sphingolipids, sterols, prenols, saccharolipids, and polyketides (29, 30). LIPID MAPS has also introduced a numerical nomenclature wherein each lipid species can be assigned a 12-digit identifier (31). A similar project has resulted in the creation of SwissLipids, a structural database detailing over 244,000 different lipids and linking them to their MS-based identifications and metabolic pathways (32). Alignment with these lipid databases will be an important part of comparing data between platforms and laboratories and essential for the establishment of clinical lipidomics platforms.

### Instrumentation

The clinical application of lipidomics necessitates that specific requirements be met if a clinical platform is to be time and cost effective. First, sample throughput must be sufficiently high, allowing an adequate number of patient samples to be processed per instrument in a given day. This is essential not only for the rapid turnaround of clinical results but also to remain cost-effective, as instrument time represents a significant contribution to the running costs of the platform. For lipidomic data to be useful, particularly in the clinical setting, it must be at least semiquantitative, providing data on lipid concentration in a reproducible manner, allowing for comparison between patients and appropriately determined reference ranges. The resolution of lipid species may also be important; in some settings, resolution of isomeric or isobaric species may be required.

MS is an ideal technique for the analysis of individual lipid species, yielding measurements of *m/z*, from which molecular mass is readily determined. By operating in tandem, such as with triple quadrupole instruments, MS/MS techniques yield a wealth of additional structural information derived from fragmentation pathways, enabling powerful structural determination. The detection limits afforded by MS techniques are exceptional, with most lipids typically sitting in the picomolar range, although specialized techniques and derivatization of compounds can increase sensitivity further. In addition, MS is well suited to direct infusion or integration with HPLC systems, allowing inline separation of compounds, thus providing orthogonal separation of chemically similar lipid species. Analysis time for both approaches is related to the number of compounds to be measured and sample complexity.

### Direct infusion

Infusion of an organic plasma extract directly into the spectrometer, without prior chromatographic separation, provides several advantages. In this approach, a greater number of experiments/transitions can be run in sequence, whereas a limited number of experiments can fit across the elution window of target compounds with chromatographic separation. As a result, direct infusion can be used to vary experimental parameters across a wide range of values and hence is often described as “shotgun” lipidomics, typically performed using an ESI-MS/MS setup (33). In addition, constant infusion of the sample results in steady analyte concentration across the entire assay, preventing variation in matrix effects, such as ion suppression. This technique has enabled the development of multidimensional MS, wherein the shotgun strategy is utilized to create multidimensional lipidomic data with alternate experiments across different axes. Typical lipidomic experiments utilized here include product ion analysis, precursor ion scanning mode, neutral-loss scanning mode, and selected reaction monitoring.

The shotgun approach has also been applied to quadrupole TOF (QqTOF) and orbitrap instruments, which offer increased mass resolution compared with triple quadrupole instruments, enabling differentiation between isobaric species. Ståhlman *et al.* (34) described a high-throughput setup, which utilizes a shotgun lipidomics approach combined with multiple precursor ion scanning experiments running on a QqTOF instrument. Their platform incorporated a 96-well plate-based automated lipid extraction system with robotic liquid handling connected to a microfluidic nanoelectrospray ionization device. They reported a sample throughput of 50 samples per day. This platform was subsequently utilized in the study of hypoxia-induced changes in the lipidome of HL-1 cells, quantifying a total of 150 lipid species. A study of analytical variation across 237 control samples, measured over a 5-day period, yielded a relative standard error (coefficient of variation) of approximately 15% throughout. A shotgun lipidomics protocol designed for clinical samples has also been published by Eggers *et al.* (35) incorporating high-resolution MS instrumentation (quadrupole-orbitrap) and the software package LipidXplorer for data analysis and quantitation.

### LC

An alternative strategy to the shotgun lipidomics approach is to incorporate in-line HPLC separation into the instrumentation setup, such that the column output is infused into the spectrometer in real time. This provides an additional dimension of characterization (specifically, column retention time). This technique has the potential to provide additional sensitivity for low abundance lipids by separating these compounds from other more abundant species. Several separation strategies are available, along with a multitude of column materials; hydrophilic interaction LC and normal phase columns find use in the fractionation of lipid classes; however, reverse phase, such as C8, C18, or C30 columns, provide a high degree of separation at the individual species level.

HPLC-MS strategies can be employed in an untargeted fashion, usually incorporating high-resolution instruments such as QqTOF and orbitrap-based spectrometers. This approach yields a large volume of lipidomic data, which are typically analyzed via comparison with *m/z* databases for known lipid species. Untargeted lipidomics has been used to study lipidome-disease associations, such as the effect of acquired obesity (36), diet-induced weight loss (37), insulin resistance (38, 39), inflammation (39), CVD (40), and response to pharmaceutical therapies (41). Such an approach is particularly useful for the discovery of new biomarkers and where the species of interest are as yet unknown but is of limited application for routine lipidomic analysis in a clinical setting.

The use of LC-MS techniques to monitor sets of predefined lipid species (targeted lipidomics) offers a number of advantages for routine clinical lipidomics, where a specific set of lipid species are known to be of interest and rapid replicable assays involving large numbers of samples are required. This approach is often used in combination with triple quadrupole mass spectrometers, typically using scheduled multiple reaction monitoring, wherein transitions are monitored during scheduled portions of the chromatographic run coinciding with elution of the lipid species of interest. This approach allows a greater number of transitions to be monitored but requires significant effort to develop; compounds of interest must be identified and their transitions, retention times, and response factors determined, some of which may change according to experimental setup. These processes involve additional offline experiments (42). Once the experimental setup is complete however, experiments can be run on large sample numbers, and the data collection, processing, and analysis can be highly automated.

### Regulatory approval of clinical platforms

Such clinical platforms will be required to meet regulatory requirements of the relevant jurisdictions of operation. While this has been achieved for most major LC platforms, the complexity and postacquisition data processing involved in MS raises some challenges to meet the transparency and auditing levels required by the Therapeutic Drug Administration in Australia or equivalent bodies in other jurisdictions. However, both the regulatory bodies and the major mass spectrometer producers are aware of these issues and are actively working to achieve regulatory approval for a number of such platforms for use in pharma, food, and health industries, with some platforms already meeting these requirements.

## A clinical lipidomics pipeline

**Fig. 3:**
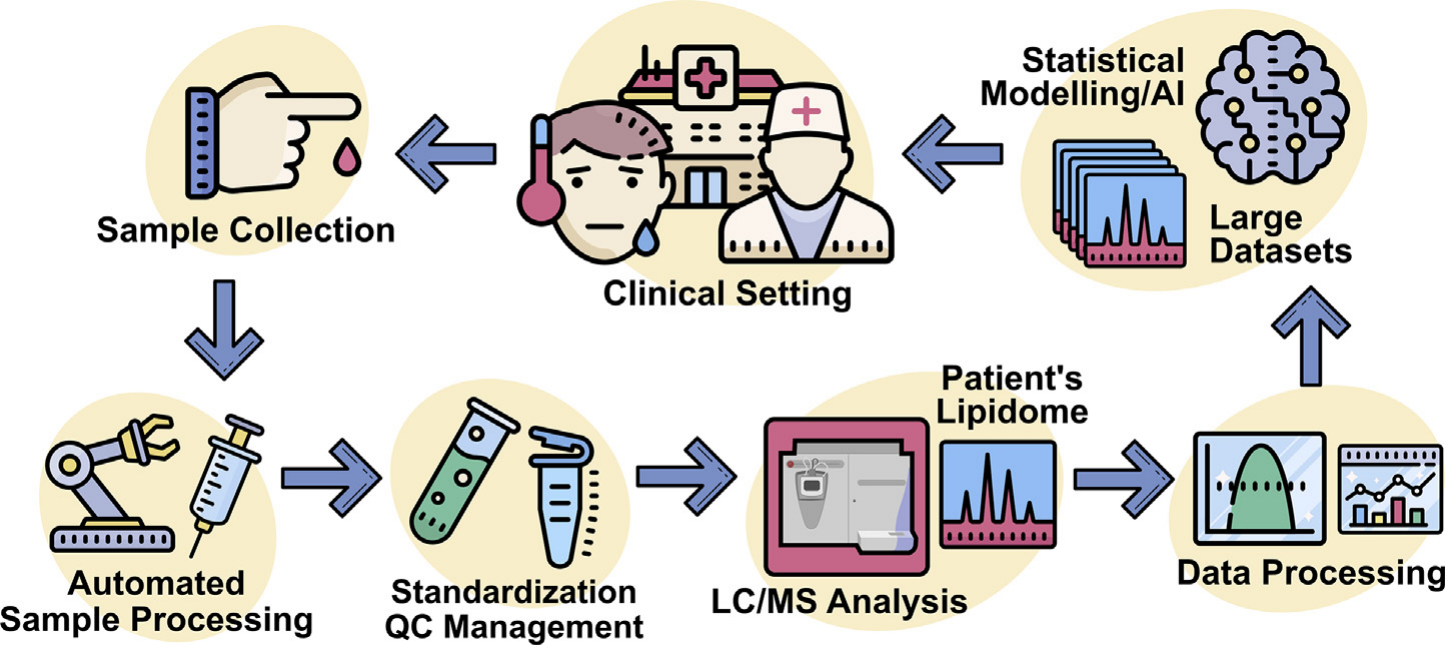
An overview of the various components that may exist within a prospective clinical lipidomic platform.

As we draw closer to the development and implementation of clinical lipidomics, it is necessary to also consider how the different parts of the analysis platform will work together in a clinical setting. An overview of various processes and components that may comprise a prospective clinical platform is provided in Fig. 3. The following discussion highlights recent developments and concepts in sample collection, sample preparation, standardization, and data processing.

### Sample collection

While lipidomics can be performed on a wide variety of biological tissues and fluids, population and clinical cohort studies typically utilize plasma or serum, as these are most readily available. There are, however, many variables that must be controlled, or at least considered, during the process of sample collection. Fasting status and even time of day at which blood is drawn can influence the lipidomic profile as demonstrated in several human circadian rhythm studies (43, 44, 45). This area may require additional investigation to determine the effect of postprandial state on the values of specific lipid species or lipidomic models. In particular, triacylglyceride species are observed to increase significantly in the postprandial period, with large individual variation. This variation in itself may prove to be a useful indicator of metabolic health (46, 47). Of course, most studies seek to measure baseline levels and reduce variation as much as possible, such that measurements taken during fasting are often the most useful.

Further variables introduced at the time of collection include the selection of anticoagulants, as variation in lipid profiles, particularly in phosphatidylcholines and triglycerides, has been observed for EDTA and citrate tubes (48). Differences in temperature and time spent on the bench, both before and after centrifugation can also impact the lipidome. Jørgenrud *et al.* (49) found that extended storage of plasma at room temperature resulted in significant lipid degradation by 24 h. It is recommended that samples are stored at −80°C, as higher temperatures (−20°C, 4°C) result in increased degradation, whereas the stability of lipids at −80°C is often considered sufficient for long-term storage and therefore clinical lipidomics. It is also recommended that freeze thaw cycles be kept to a minimum, ideally less than 2 (50, 51).

While it is standard to collect a significant volume of blood for clinical analyses, with typical collection tubes ranging 2–9 ml, this typically requires the patient to attend a doctor's surgery or pathology clinic and the services of a health provider. However, as we move toward clinical lipidomics and the concept of personalized health, more convenient and less invasive methods of sample collection may be considered. One such method currently under investigation is the use of dried blood spots, which are easier to collect and can potentially be performed by individuals in their own home (52, 53). Koulman *et al.* (54) reported on the measurement of 56 different lipids, including cholesteryl esters, phosphatidylcholines, sphingomyelins, and triglyceride species in dried blood spots from infants and found that although oxidized lipids and hydrolysis products were clearly detectable, they were of minimal impact to the overall lipid profile. Interestingly, experimental precision was equal to that of a traditional larger volume sample collection.

### Sample preparation

To submit samples for mass spectrometric analysis, whether chromatographic separation is employed or not, lipids must first be extracted from their native state–lipoproteins in the context of plasma or serum samples—and into a suitable organic solvent. While this process can be automated, it provides a potential source of error and mandates the introduction of additional quality control measures. A variety of extraction techniques and solvents have been employed in the past, including protocols by Bligh and Dyer (55) or Folch *et al.* (56), which use two-phase extraction techniques and chloroform/methanol/water solutions. One issue presented by these methods is the high volatility of chloroform solutions, as evaporation during handling is a potential source of experimental error, sometimes avoided by additional evaporation/reconstitution steps. Collection of the lower organic phase in two-phase liquid-liquid extractions can also be cumbersome.

To avoid these issues, a method using methyl-tert-butyl-ether has been developed by Matyash *et al.* (57), as methyl-tert-butyl-ether forms an upper organic phase for easier collection. However, such two-phase systems can result in partitioning of some lipid species between phases and incomplete recovery. In our own laboratory, we developed a single-phase protocol using butanol/methanol, which eliminates the use of more volatile solvent systems, and requires no drying or reconstitution steps (58, 59). The butanol/methanol protocol appears to provide equal recovery of most lipid species, with improved extraction of polar lipids (60). One potential drawback of such single-phase methods is the less efficient removal of unwanted ionic compounds, which precludes its use in direct infusion approaches and may be particularly relevant for high-throughput applications. Researchers should consider the trade-offs between purification efficiency and the recovery of more polar lipids when selecting one-phase or two-phase extraction protocols.

### Standardization

To facilitate the quantitation of MS data and account for variation in extraction recovery, matrix effects as well as ionization and fragmentation efficiencies, it is usual to include a series of internal standards (ITSDs) that are added prior to extraction. Lipid concentration can then be determined by normalizing the lipid signal to a class-specific or subclass-specific ITSD. Ideally, a stable isotope labeled ITSD would be used for each lipid species analyzed; however, this is often prohibited by cost and availability, as the number of lipids in clinical studies may number in the hundreds. Neither is this possible for untargeted research, where the compounds of interest are not known at the time of analysis. Thus a single or several ITSD compounds are typically employed for each lipid class. This approach has been found to be sufficiently quantitative for cohort studies where precision is more important than absolute accuracy for subsequent association analyses (61, 62) but may not be sufficient for a clinical lipidomics platform.

An additional strategy, typically adopted for batch correction, is to include a quality control sample spaced regularly throughout the analyses of large multibatch experiments, which is then used to standardize each lipid species across batches and thereby reduce variation within the experiment. This approach has now been demonstrated to also perform well between platforms and laboratories to align results (63) and offers a path forward for clinical lipidomics. The National Institute of Standards and Technology (NIST) Standard Reference Material (SRM) 1950 has been proposed as a suitable reference material for clinical platforms as it has been extensively analyzed and is commercially available.

In a recent ring trial led by the NIST, Bowden *et al.* coordinated the measurement of over 1,500 unique lipid species from the NIST SRM 1950—Metabolites in frozen human plasma. Thirty-one laboratories participated in the study, which provided a consensus estimated for 339 lipid species, measured in at least five laboratories. The study found phospholipids to be measured more consistently compared with other species with approximately 52%, 48%, 34%, and 80% of lysophosphatidylcholine, PC, phosphatidylethanolamine, and phosphatidylinositol species returning coefficient of dispersion values of less than 20%. However, the study also highlighted the variation between laboratories in measuring the lipid species with coefficient of dispersion values ranging from 10% to over 80% across the entire lipid panel (64). If we are to progress toward clinical lipidomics, then it will be necessary to address the quality control issues highlighted in the NIST analysis.

### Data processing

Regardless of the specific experimental protocols, lipidomics performed in a clinical setting will necessarily be high throughput, resulting in the generation of large volumes of data requiring further processing to extract useful information. As yet, there is no software solution that provides a complete level of automation for “on-the-fly” data processing suitable for a clinical lipidomics platform. There are however a number of advanced software solutions available, and this space is developing at an increasing pace.

For shotgun lipidomics and multiple precursor ion scanning (spectra), a number of software packages exist to facilitate data processing. The Lipid Profiler and Lipid View (MDS Sciex) (34, 65, 66) packages achieve lipid identification through comparison with a database containing information on the precursor and fragment ion *m/z* of various lipid species. The software is in addition able to perform corrections to quantify lipid precursors where there is isotopic overlap. For LC-MS, Masshunter MS Quantitative Analysis software (Agilent Technologies) is effective in the batch processing of multiple reaction monitoring data, providing chromatogram peak areas in an automated fashion. The package includes peak picking algorithms that automate integration of chromatograms allowing for rapid data processing.

*Lipidr* is an open-source R-based package, which allows importation of lipidomic data into an R-based environment with automated interpretation of lipid class and chain length from the provided names. The package allows for data inspection, normalization, univariate and multivariate analyses, and visualization (67). Indeed, many laboratories typically use a suite of commercially available software and custom scripts to assist in analysis of clinical samples. Ultimately, a solution that incorporates peak integration, internal standards, and reference material standardization in a real-time analyses will likely be developed on a platform-specific basis as the field progresses.

## Future pathways

### Regulatory requirements

Subsequent to technical development and validation, prospective clinical lipidomics platforms would typically require accreditation from the relevant regulatory body in the country of use. Within Australia, laboratory accreditation is governed by the National Association of Testing Authorities and is awarded subsequent to the ongoing assessment of laboratory management systems, staffing and location, methods and validation, quality control systems, equipment and calibration, as well as the reporting systems. Accreditation of a prospective clinical platform itself requires registration as an in vitro diagnostic device with the Australian Therapeutic Goods Administration, wherein it would be assigned one of four classes according to its perceived personal and public health risk. Said classes determine the level of assessment carried out; in the case of a diagnostic test to assist in the diagnosis and treatment of serious disease, we anticipate that prospective clinical platforms would be assigned as class 3 in vitro diagnostic devices (moderate risk). Accordingly, manufacturers would need to undergo strict conformity assessment to ensure the device meets quality and safety requirements, and an essential principles review, including relevant published literature and technical documentation.

Most assays incorporating the measurement of specific chemical species would seek to demonstrate analytical accuracy and precision. For the clinical lipidomics platform, we anticipate some challenges may arise from the incorporation of an increasingly large number of lipid species, some of which may only be measured semiquantitatively. We envision that rather than seeking approval for each individual lipid assay, the postmodeling metabolic risk score would be the focus of registration and thus analytical scrutiny.

### Clinical utility

Prospective clinical lipidomics platforms must demonstrate their clinical utility; that is, their ability to inform and impact patient diagnosis and care. Prior to device registration, such assessment would be carried out as part of the validation process, likely using sample sets from clinical trials and seeking to demonstrate significant risk restratification compared with other measures. However, such assessment would need to be carried over to real-world applications and include postmarket monitoring and reporting to allow ongoing assessment within the clinical setting.

### Research initiatives

As analytical technology continues to advance, we will likely see the incorporation of an increased number of lipid measures in future clinical protocols. Furthermore, sample throughput in prospective clinical lipidomics platforms will climb sharply upon their deployment in clinical and pathology settings. Accordingly, cooperation across the international research community will become increasingly important, particularly for the standardization of technical practices and analytical measures between laboratories. To this end, the International Lipidomics Society has been established to foster international collaboration and the development of new technologies, skills, and resources in the lipidomics field. The aforementioned projects such as LIPID MAPS and SwissLipids are also playing an important role. Recent progress in this area of broad international collaboration includes the publication from Thompson *et al.* of an international ring trial from 14 laboratories, in which they quantified up to 408 metabolites in blood specimens using a shared experimental protocol. The study provided another demonstration of the utility provided by the NIST SRM 1950 reference sample, with the finding that with the appropriate controls, high levels of accuracy and precision can be achieved across laboratories. The authors describe a system suitability testing protocol to evaluate the capacity of a given laboratory to participate in the trial, including factors such as signal abundance, mass accuracy, retention time, and peak shapes, with the data published in a public repository (68). These and other community-led initiatives will be important contributors to the development of clinical lipidomics.

## Conclusions

The advent of modern lipidomic profiling techniques has led to significant advances in our understanding of the fundamental biological processes involved in health and disease. This technology has now reached the point at which high-throughput sample preparation and batch analysis enable its application to large cohort clinical trials, generating huge volumes of data that capture the breadth of metabolic conditions present in the human population. Significant effort is currently underway to mine this data for new associations and biomarkers, pinpointing the fundamental processes involved in disease etiology, as well as discovering additional biomarkers to aid in diagnosis.

At present, many applications of lipidomic profiling remain confined to the research laboratory; however, this looks set to change. The ongoing development of advanced risk modeling procedures means that this technology now has significant application within the clinical environment, with the potential to assist in the routine diagnosis of disease, to stratify risk postdiagnosis, and to monitor the efficacy of both pharmaceutical and lifestyle interventions. The task now ahead of us is to implement this technology in an impactful way such that it becomes an accessible and effective tool for health care providers.

## Conflict of interest

The authors declare that they have no conflicts of interest with the contents of this article.
